# Volatile organic compounds (VOCs) in exhaled breath as a marker of hypoxia in multiple chemical sensitivity

**DOI:** 10.14814/phy2.15034

**Published:** 2021-09-18

**Authors:** Andrea Mazzatenta, Mieczyslaw Pokorski, Camillo Di Giulio

**Affiliations:** ^1^ Department of Neuroscience, Imaging and Clinical Sciences University “d’Annunzio” Chieti‐Pescara Chieti Italy; ^2^ Institute of Health Sciences University of Opole Opole Poland; ^3^ Faculty of Health Sciences The Jan Dlugosz University in Czestochowa Czestochowa Poland

**Keywords:** hyperventilation, hypoxia, lactate, multiple chemical sensitivity, ORT test‐VOCs, VOCs

## Abstract

In the history of diagnostics, breath analysis was one of the first method used until the breakthrough of biochemical testing technology. Today, breath analysis has made a comeback with the development of gas analyzers and e‐noses, demonstrating its power in its applicability for diagnosing a wide range of diseases. The physical basis of multiple chemical sensitivity (MCS), an emerging environmental disease, is difficult to understand because it is based on the scenario of chronic hypoxia, with a complex of chemical compounds that trigger the syndrome and result in multiple symptoms.

The aim of this study was to investigate MCS by analyzing exhaled volatile organic compounds (VOCs). The volatile, metabolic picture could be a putative gold standard for understanding and diagnosing the disease. The study was based on recording in resting condition using the noninvasive passive e‐nose contactless breath test, the Olfactory Real‐Time Volatile Organic Compounds (ORT‐VOC) test in MCS, and control samples. The VOCs profile distinguished between disease and health. It also distinguished the gender‐related volatile profile with significant robustness. The results trace a putative compensatory physiological pathway elicited by increased lactate, leading to acidosis, and hyperventilation, resulting in the production of specific VOCs. We conclude that breath testing is a valuable tool to investigate the hypoxia‐related VOC profile, facilitating MCS diagnosis.

## Graphical abstract

MCS results in a compensatory physiological pathway elicited by the increase in lactate, leading to acidosis and hyperventilation. ORT‐VOC breath test is a valuable tool to investigate the hypoxia‐related VOC profile. ORT‐VOC breath test is facilitating the MCS diagnosis.

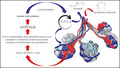

## 1 INTRODUCTION

Endogenous volatile organic compounds (VOCs) are produced during catabolism in the body. These gasses pass from tissues and organs into the bloodstream and are ultimately exchanged in the lungs and exhaled. Exhaled VOCs provide a unique source of useful biomarkers with direct associations to the body's metabolism (Mazzatenta et al., 2013).

Ancient Greek physicians diagnosed diabetes by the sweet, fruity smell of acetone breath, liver disease by the smell of musty and fishy reek, kidney failure by the urine‐like smell, and lung abscess by the putrid stench (Phillips, 1992). Nowadays, descriptions of the composition and characteristics of VOCs human breath have increased greatly. A large number of breath markers have been identified in a number of diseases due to systemic inflammation that alters the composition of the exhalate (Mazzatenta, Di Giulio, et al., 2013). Consequently, biopsy and analysis of breath VOCs have been improved and used as noninvasive clinical tools for disease diagnosis and monitoring (Di Gilio et al., 2020). Exhaled VOCs produced under the influence of oxidative stress and inflammatory response are useful to investigate the physiological mechanism of an emerging environmental disease called multiple chemical sensitivity (MCS). It is characterized by phobosmia and hyperosmia frequently related to a toxicant‐induced loss of tolerance that develops in response to acute toxic exposure or cumulative effects of non‐threshold low‐dose and long‐term acting contaminants (Bartha, 1999; Lanphear, 2017; Mazzatenta, Pokorski, et al., 2013). Most patients with MCS are women having poly‐symptomatic complaints.

MCS could be described as hypoxic, oxidative stress, and inflammatory disease due to sensitization to N‐methyl‐D‐aspartate (NMDA), increased peroxynitrite and nitric oxide, manifestations of pro‐inflammatory cytokine, decreased glutathione, altered redox enzymes, and cytochrome P450 metabolism, altered serotonin receptors, neural sensitization, and neurogenic inflammation (Gugliandolo et al., 2016; Mazzatenta, Pokorski, et al., 2013; Pigatto et al., 2013). Since its first description in 1956 and subsequent attempt to define it in 1987, MCS has been the subject of great criticism due to an uncertain physiological basis and a challenging diagnosis (Cullen, 1987a; Randolph, 1961). Above all, MCS is characterized by adverse health effects related to, or exacerbated by, exposure to chemicals. The most common chemical triggers of MCS are blended perfumes but not pure odorants (Ross et al., 1999). Changes in latencies of chemosensory event‐related potentials indicate altered processing of both olfactory and trigeminal stimuli by MCS, increasing the susceptibility of potentials to volatile environmental chemicals (Hummel et al., 1996).

Odors can act as a warning of a potential health risk as they can be detected at much lower concentrations than molecules that irritate the respiratory tract. Consequently, the confusion between odorants and irritation coupled with variabilities in the sensitivity and responses to odors are significant obstacles in the assessment of MCS (Dalton, 2003). MCS patients show stronger responses to threshold olfactory stimulation in the prefrontal cortex. Interestingly, the response is associated with a decrease and delayed recovery of peripheral blood SpO_2_ compared to that of controls. Changes in SpO_2_ suggest that MCS might result in tissue hypoxia and metabolic acidosis, which often is confirmed by clinically observed hyperventilation with a rapid fall in PCO_2_ either no change or rise in PO_2_ (Azuma et al., 2013, 2018; Leznoff, 1997). Consistently with tissue hypoxia, MCS is associated with shortness of breath, depression, anxiety, chronic fatigue, and fibromyalgia‐like muscle pain.

In the scenario outlined above, exhaled VOCs in MCS patients could be taken as a putative marker of hypoxia. Therefore, this study aims to investigate the potential presence of characteristic features in the exhaled VOC profile that could become the basis for distinguishing between the disease and the norm.

## 2 MATERIALS AND METHODS

This is a retrospective study following the Declaration of Helsinki and the Standards and Operational Guidance for Ethics of Health‐Related Research with Human Participant (World Health Organization, 2011; World Medical Association, 2008). Anonymous data of 50 subjects, who underwent the Olfactory Real‐Time Volatile Organic Compounds (ORT‐VOC) breath test were grouped in 25 healthy controls (7 males—mean age of 48.7 ± 9.3 SD years, range of 32–61 years and 18 females—mean age of 45.4 ± 11.1, range of 30–63) and 25 MCS patients (6 males—mean age of 47.8 ± 10.2 SD years, range 32–60 years and 19 females—mean age of 47.4 ± 9.7, range of 30–65). Patients fulfilled the international criteria for the diagnosis of MCS disorder (Bartha, 1999; Cullen, 1987b). ORT‐VOC breath recordings were made using an e‐nose sensor (iAQ‐2000; Applied Sensor) according to the standard analytical method [for details see 6, 20–23].

Raw data were transformed and normalized using the formula (1/log10X)^y^ and presented as means ± SD and analyzed using MatLab, Jamovi, Origin software. Statistical analysis of intergroup differences was performed with MANOVA and one‐way ANOVA post‐hoc tests, with the alpha level of 0.001 defining the significance of differences.

## 3 RESULTS

### 3.1 MCS versus control exhaled VOCs

**FIGURE 1:**
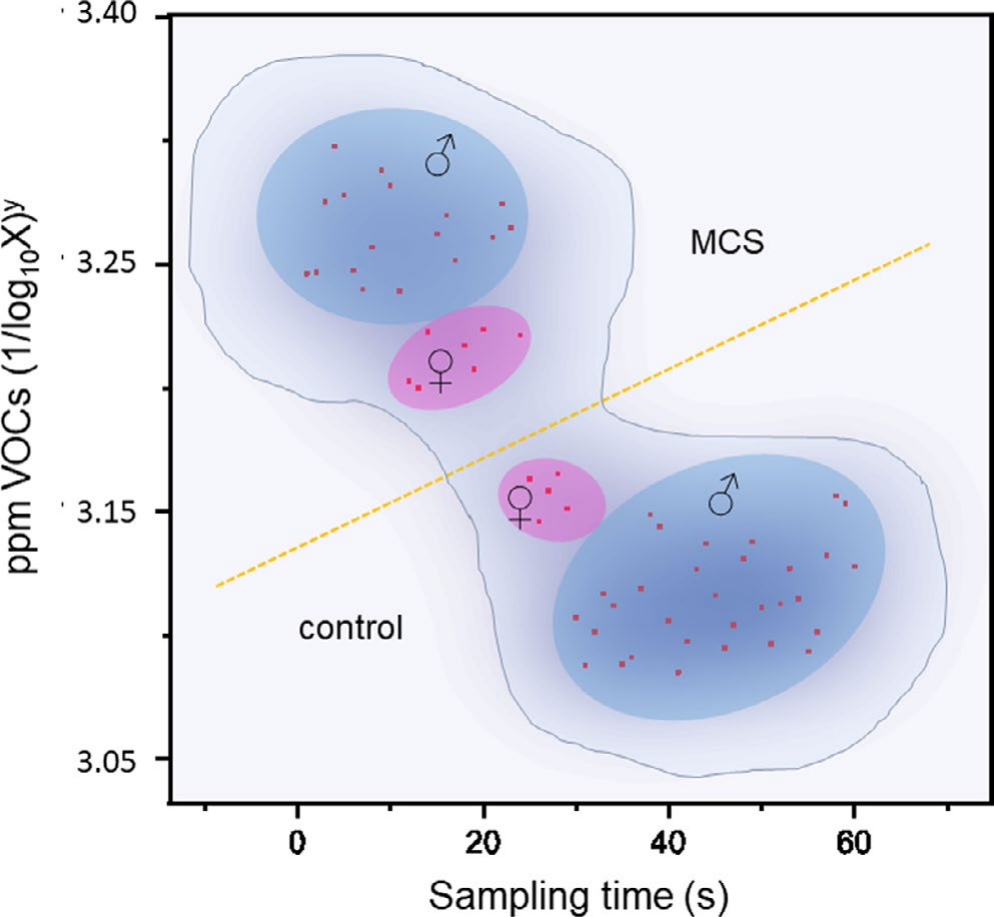
The ORT‐VOCs test shows a clear separation of data between the MCS and control groups, and between the sexes

Basal exhaled VOCs data for MCS and controls, acquired with the ORT‐VOC, are shown in a density plot (Figure 1). The data were also segregated according to the sex of the participants. A wider spread of density in male participants results from a limited sample size. The MANOVA analysis showed significant differences between the MCS and controls (*p* < 0.001, F_(1,118)_ = 7563.39; mean for MCS of 3.25 ± 0.007 SD ppm; mean for controls of 3.14 ± 0.006 SD ppm). VOCs analysis robustly discriminated exhaled breath in disease subjects compared to controls in real‐time, *p* < 0.001.

### 3.2 Exhaled VOCs depending on the sex

**FIGURE 2:**
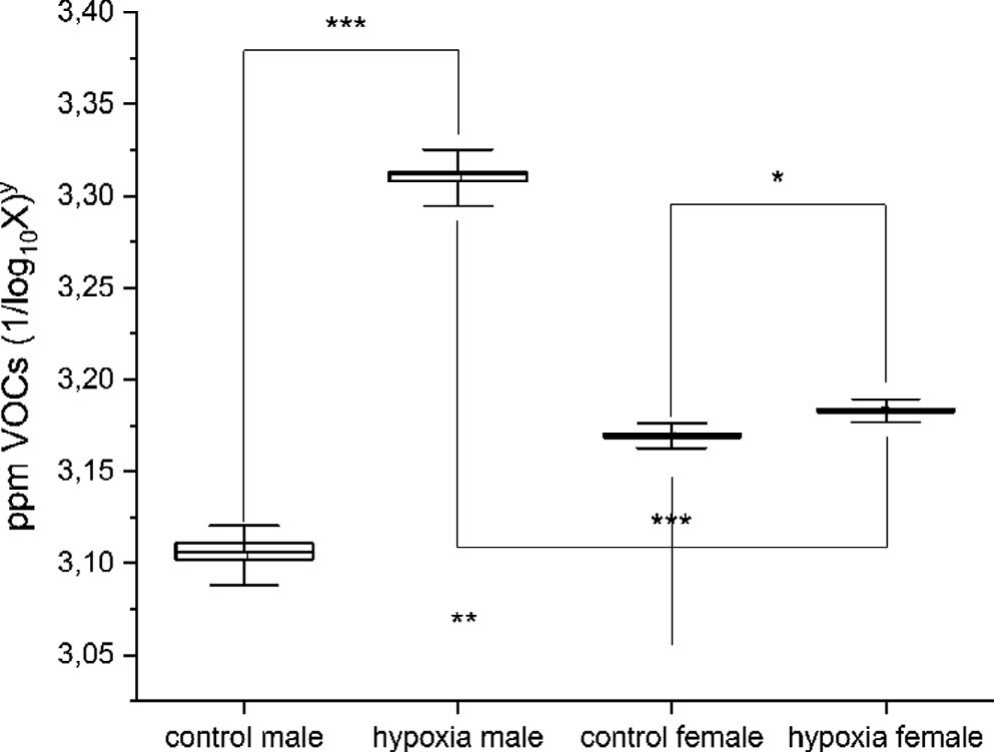
Sex‐dependent differences in the ORT‐VOC data in MCS and control groups. Box and whiskers plots. Differences were significant both within and between groups: *p < 0.05; **p < 0.001; ***p<<0.001

Figure 2 shows sex‐dependent differences in ORT‐VOC tests results. A post‐hoc one‐way ANOVA pointed to significant differences between male and female participants within both MCS and control groups, *p* < 0.001 (MCS sex F_(1,118)_ = 3525.99; mean MCS male of 3.31 ± 0.015 SD ppm; female of 3.18 ± 0.006 SD ppm; MCS control F_(1,118)_ = 842.22; mean control male of 3. 1 ± 0.016 SD ppm; female 3.17 ± 0.007 SD ppm). In addition, significant sex differences were present between the MCS and control groups, *p* < 0.001 (male MCS vs. control F_(1,118)_ = 5165.5; female MCS vs. control F_(1,118)_ = 132.6). Consequently, VOC analysis was able to recognize sex in both healthy and sick participants.

### 3.3 VOCs frequency and principal component analysis

**FIGURE 3:**
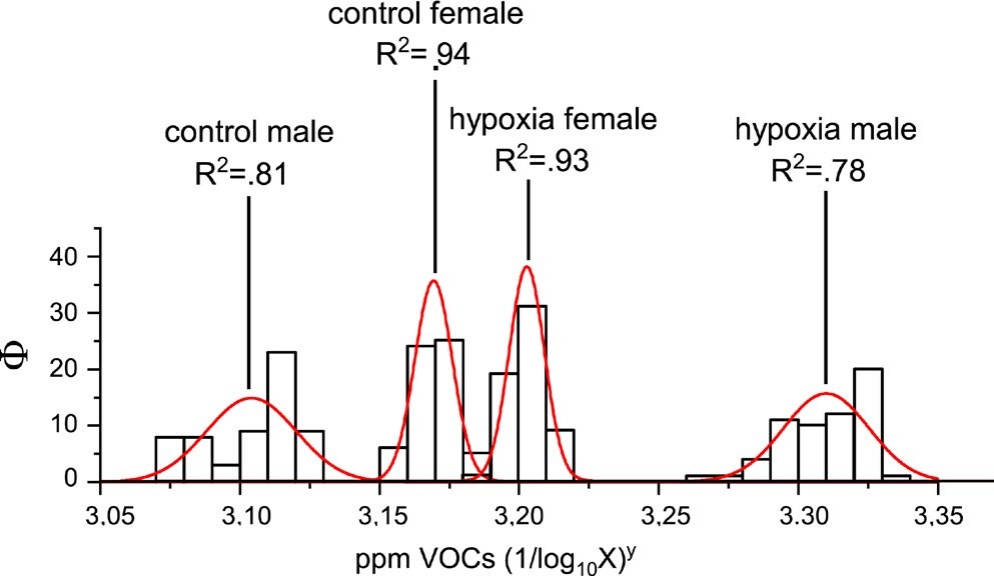
VOCs frequency analysis and distribution fit per sex in MCS and control groups

VOCs frequency was plotted as a histogram grouped by sex in MCS versus controls (Figure 3). A fit for each sex group showed a normal distribution with highly significant MCS male R^2^ = 0.78 and female R^2^ = 0.93, and control male R^2^ = 0.81 and female R^2^ = 0.94. As aforementioned¸ a more dispersed distribution in males is related to smaller sample size.

**FIGURE 4:**
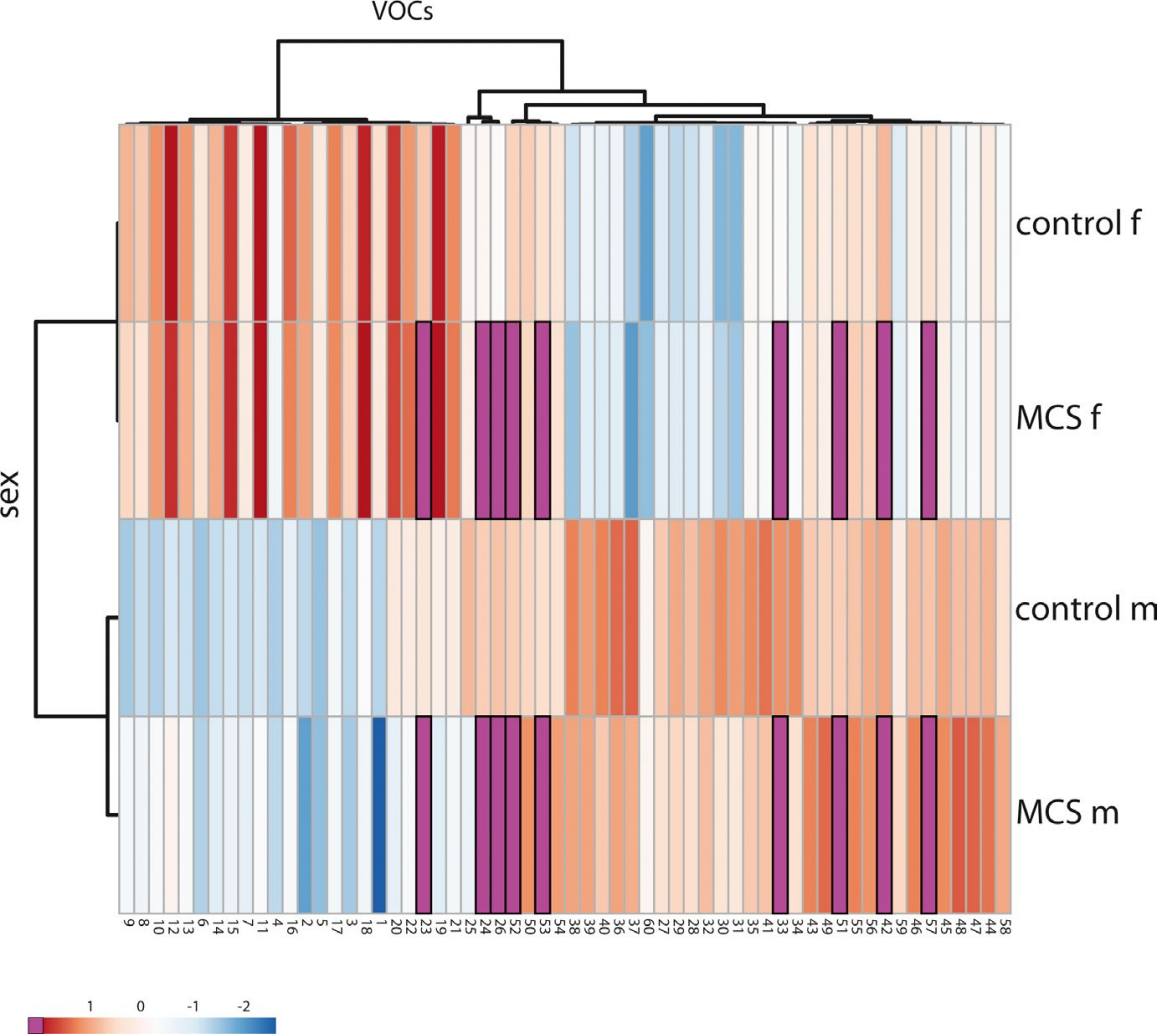
Principal component analysis showing clustering of sex (light blue and tan) and MCS (purple bars) disease‐related characteristics

Principal component analysis showed characteristic clustering related to sex and MCS disease (Figure 4). The analysis also revealed partially different and converging VOC characteristic component profiles in both MCS and control subjects of both sexes.

## 4 DISCUSSION

Since Pauling discovered that humans exhale a large number of VOCs in low concentrations (Pauling et al., 1971), it has enabled the understanding of numerous physiological processes (Sinues et al., 2013), such as oxidative stress (Aghdassi & Allard, 2000), and the identification of new disease biomarkers (Beccaria et al., 2018; Mazzatenta, Di Giulio, et al., 2013; Schleich et al., 2016). Pauling's techniques usually reveal around 70 different VOCs in a breath sample, today the methods for collecting, concentrating, separating, and identifying breath VOCs have progressively improved over the last few decades to comprehensive two‐dimensional gas chromatography. Comprehensive two‐dimensional gas chromatography, and its innovations, is currently the gold standard for VOC profiling, with these improvements an increasingly complex picture of the composition of breath VOCs has emerged in excess of thousands of compounds (Das et al., 2014; Lacy Costello et al., 2014; Mansurova et al., 2018; Phillips et al., 2013). Consequently, the compendium of all VOCs emanating from the human breath and body, the Volabolome, is steadily increasing and includes hydrocarbons, alcohols, ketones, and aldehydes at levels from ppb to ppm, putatively much more at less concentration will be discovered. For instance, the major ubiquitous VOCs in the breath of healthy individuals are isoprene (12–580 ppb), acetone (1.2–1880 ppb), acetaldehyde (3–7 ppb), ethyl acetate (ND‐116 ppb), butanone (6–26 ppb), 1‐butene (ND‐495 ppb), dimethyl sulfide (ND‐46.5 ppb), ethylene (ND‐233 ppb), furan (ND‐78.4), hexanal (9–13 ppb), methyl ethyl ketone (ND‐45.3 ppb), ethanol (13–1000 ppb), methanol (160– 2000 ppb), isopropanol (50–260 ppb), and other alcohols, while minor components include pentane (14–43 ppb) and higher aldehydes and ketones (Wilde et al., 2019; Zhou et al., 2019).

Exhaled breath contains a mixture of volatile metabolites that can provide an integrated view of the upstream pathophysiological processes for which the term Volabomics has been recently coined (Mochalski et al., 2013). The gold standard for profiling the range of different VOCs and understanding which of them may be representative of MCS cases and useful for discriminating between MCS and other related disorders is comprehensive two‐dimensional gas chromatography (Das et al., 2014; Lacy Costello et al., 2014; Mansurova et al., 2018; Phillips et al., 2013). Exhaled gas biopsy enables noninvasive collection and analysis of VOCs (Giannoukos et al., 2019) till the analysis of cell cultures (Baranska et al., 2015) By sampling the breath for merely 1 min, which is roughly the time of blood flow over the circulatory system, incoming VOCs from all parts of the body are detected (Baldwin et al., 1986). The ORT‐VOC test, based on the e‐nose device, has been useful to analyzing exhaled VOCs in real‐time in several diseases (Invitto & Mazzatenta, 2019; Mazzatenta et al., 2014, 2015, 2016; Mazzatenta, Pokorski, et al., 2013). The method is also useful in patients with MCS who have a reduced quality of life due to limitations in daily activities and household chores (Bell & Baldwin, 2013; Mazzatenta, Pokorski, et al., 2013).

MSC is a chronic progressive condition whose full definition is yet to be settled. The disease involves multiple body systems with central nervous sensory disturbances such as hyperosmia and phobosmia, and musculoskeletal, respiratory, and gastrointestinal symptoms.

The MCS share significant homology and overlap with several environmental diseases and intolerances, for example, Gulf War Illness, multi‐sensory sensitivity, chronic fatigue syndrome, fibromyalgia, sick building syndrome, irritable bowel syndrome or inflammatory bowel disease, e‐cigarette, or Vaping, product use Associated Lung Injury (EVALI). In particular, the relevant common symptoms are systemic inflammation, impaired oxidative metabolism, chronic oxidative stress, mitochondrial dysfunction, higher blood lactate, and tissue hypoxia associated with frequent headaches, shortness of breath, depression, anxiety, chronic fatigue, and muscle pain (Cordero et al., 2010; Donnay & Ziem, 1999; Efrati et al., 2015; Johnson et al., 2016; Lacoura et al., 2005; Landi et al., 2016; Manczak et al., 2005; Meeus et al., 2013; O’Donovan et al., 2015; Parkitny et al., 2015; Proia et al., 2019; Shetty et al., 2017; Van Malderen et al., 2020).

Studies on MCS and related diseases show that pH regulation is a physiological factor to be taken into account, because acidification due to an increase in CO_2_ inducing an acute production of reactive oxygen species stimulates the synthesis of hypoxic‐inducible factor 1 alpha (Wanandi et al., 2021). It affects aerobic glycolysis which occurs when O_2_ availability is sufficient to create large amounts of lactic acid as an end product, similar to the Warburg effect (Damaghi et al., 2013). Lactate production due to the acidic environment reduces oxygen levels leading to hypoxia, which upregulates the stress‐induced reaction, a putative physiological mechanism of MCS. Presumably, MCS is underlain by hypoxia/hypercapnia (H/H) due to disturbed respiration (Mazzatenta, Pokorski, et al., 2013; Ross, 2000; Rossi & Pitidis, 2018). Interestingly, EVALI is characterized by an elevated lactate dehydrogenase (1028 U/L), which putatively resembles an acute form of MCS (Cobb & Abrams, 2011).

Thus, a link emerges between increased lactate and homeostatic response to acidification that increases VOCs emission. Consistently, we show in the present study that the increase in VOCs is a sensitive discriminator of MCS patients from control subjects of both sexes. This outcome is of novel value for gaining deeper insights into the MCS pathophysiology as we provide evidence of an organic basis of the syndrome. Additionally, we show that the VOCs assessment of exhaled breath differs depending on the patient's sex. In a previous study, we showed an increase in VOCs in response to poor inhaled air quality and impending hypoxic conditions (Mazzatenta, Pokorski, et al., 2013).

Thus, the biological plausibility of MCS induction arises from a cross‐over interaction between the NO/ONOO^−^ vicious cycle and the oxygen supply cascade linked in two ways directly via NO and indirectly via the oxidative stress‐mediated release of ROS that causes neural sensitization through activation of multiple NMDAs (Mazzatenta, Pokorski, et al., 2013; Pall et al., 2009). The key steps would be the formation of NO and ROS, both of which increase NMDA receptor activity, which creates the basis for overlapping effects of exposure to pollutants or hypoxia that evoke MCS symptoms (Mazzatenta, Pokorski, et al., 2013; Pall et al., 2009; Wanandi et al., 2021).

**FIGURE 5:**
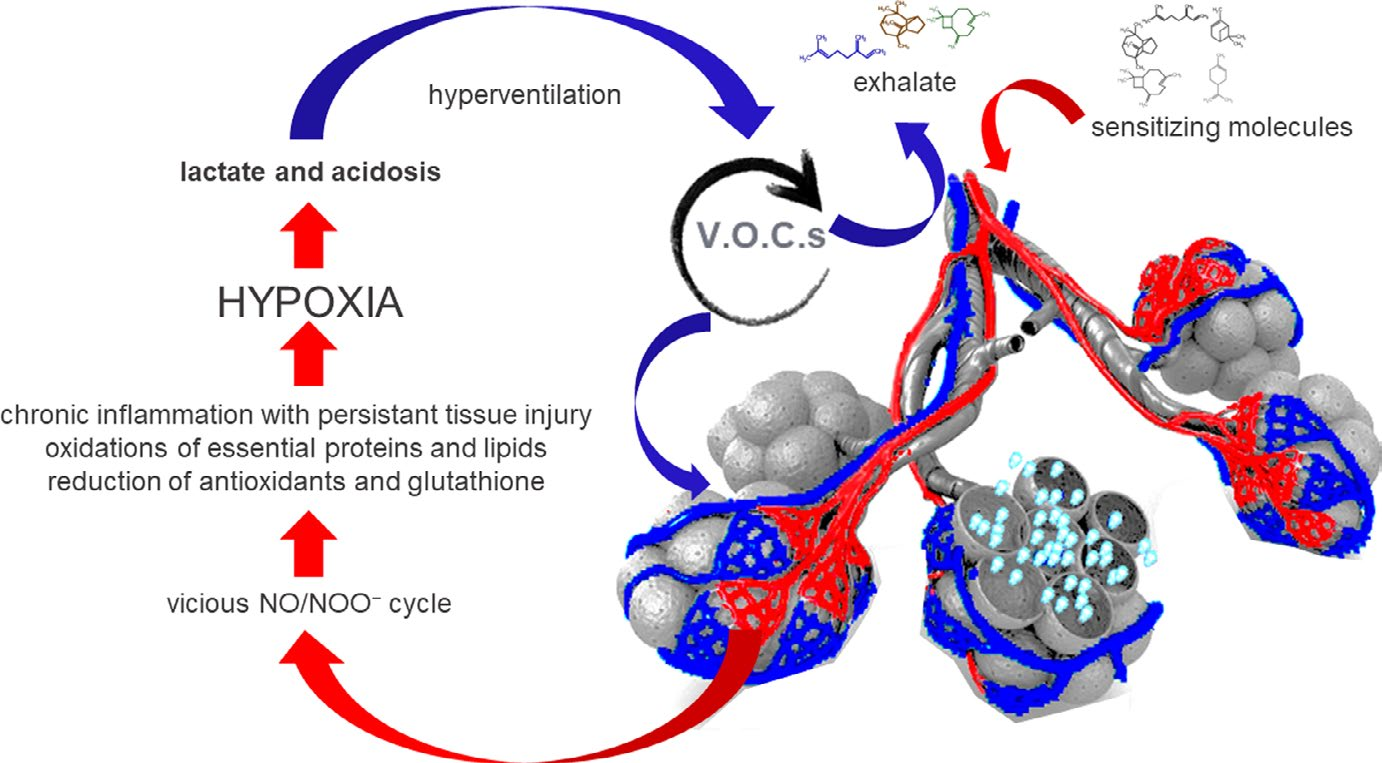
Exhaled VOCs are gasses exchanged at the lungs released to the bloodstream, produced from tissues and organs catabolism. In MCS, sensitizing molecules activate vicious NO/NOO^−^ cycle facilitating lactate production and body hypoxia leading to the development of acidosis and lung compensatory hyperventilation, resulting in the formation of a characteristic exhaled VOC profile

The putative pathways elicited by sensitizing molecules in the activation of vicious NO/NOO^−^ cycle, stimulating lactate production and tissue hypoxia leading to acidosis and compensatory pulmonary hyperventilation, and eventually form specific exhaled VOCs profiles that we recorded in the present study and are graphically presented in Figure 5.

We conclude that further studies on a larger number of subjects with MCS and closely related disorders should be carried out to fully understand the diagnostic potential of breath analysis. Due to the characteristic pattern of MCS that falls within the forms of hypoxia. Exhaled breath tests ranging from comprehensive two‐dimensional gas chromatography to e‐noses and new emerging technologies are used to assess VOCs profiles, which has the potential to become valuable tools in the diagnosis of MCS, and other diseases.

## CONFLICT OF INTEREST

The authors declare no conflict of interest.

## AUTHOR CONTRIBUTIONS

Conceptualization, methodology, data curation, formal analysis, figure preparation, writing original draft preparation A.M.; validation A.M. C.diG., and MP; writing review and editing M.P.; supervision C.diG..

## INSTITUTIONAL REVIEW BOARD STATEMENT

The study was conducted according to the guidelines of the Declaration of Helsinki, ethical review and approval were waived for the retrospective nature of the study that was based on the analysis of anonymized data collected during routine diagnostic otolaryngological examinations.
